# Tuberculosis among Children and Adolescents at HIV Treatment Centers in Sub-Saharan Africa

**DOI:** 10.3201/eid2612.202245

**Published:** 2020-12

**Authors:** Anna M. Mandalakas, Alexander W. Kay, Jason M. Bacha, Tara Devezin, Rachel Golin, Katherine R. Simon, Dilsher Dhillon, Sandile Dlamini, Andrew DiNardo, Mogo Matshaba, Jill Sanders, Lineo Thahane, Pauline M. Amuge, Saeed Ahmed, Moorine P. Sekadde, Neway G. Fida, Bhekumusa Lukhele, Nodumo Chidah, David Damba, Joseph Mhango, Moses Chodota, Makhorong Matsoso, Angelina Kayabu, Richard S. Wanless, Gordon E. Schutze

**Affiliations:** Texas Children’s Hospital, Houston, Texas, USA (A.M. Manadalakas, A.W. Kay, J.M. Bacha, T. Devezin, K.R. Simon, D. Dhillon, S. Dlamini, A. DiNardo, M. Matshaba, J. Sanders, L. Thahane, S. Ahmed, N. Chidah, D. Damba, M. Chodota, M. Matsoso, A. Kayabu, R.S. Wanless, G.E. Schutze);; Baylor College of Medicine, Houston (A.M. Manadalakas, A.W. Kay, J.M. Bacha, T. Devezin, D. Dhillon, A. DiNardo, B. Lukhele, R.S. Wanless, G.E. Schutze);; Baylor College of Medicine Children’s Foundation Swaziland, Mbabane, Swaziland (A.W. Kay, S. Dlamini, B. Lukhele);; Baylor College of Medicine Children's Foundation Tanzania, Mbeya, Tanzania (J.M. Bacha);; US Agency for International Development, Washington, DC, USA (R. Golin);; Baylor College of Medicine Children’s Foundation Malawi, Lilongwe, Malawi (K.R. Simon, S. Ahmed, J. Mhango);; Technical Support to PEPFAR Programs in the Southern Africa Region, Lilongwe (K.R. Simon, S. Ahmed, J. Mhango);; Botswana-Baylor Children’s Clinical Centre of Excellence, Gaborone, Botswana (M. Matshaba, N. Chidah);; Baylor College of Medicine Children’s Foundation Lesotho, Maseru, Lesotho (J. Sanders, L. Thahane, M. Matsoso);; Baylor College of Medicine Children’s Foundation Uganda, Kampala, Uganda (P.M. Amuge, D. Damba);; National Tuberculosis and Leprosy Programme, Kampala, Uganda (M.P. Sekkade);; US Agency for International Development, Pretoria, South Africa (N.G. Fida);; Baylor College of Medicine Children’s Foundation Tanzania, Mwanza, Tanzania (M. Chodota, A. Kayabu)

**Keywords:** tuberculosis, HIV, antiretroviral therapy, epidemiology, adolescents, children, HIV/AIDS and other retroviruses, bacteria, viruses, respiratory infections, tuberculosis and other mycobacteria, Botswana, Eswatini, Lesotho, Malawi, Tanzania, Uganda, Mycobacterium tuberculosis

## Abstract

HIV-infected children and adolescents are at increased risk for tuberculosis (TB). Antiretroviral therapy (ART) reduces TB risk in HIV-infected adults, but its effectiveness in HIV-infected children and adolescents is unknown. We analyzed data from 7 integrated pediatric HIV/TB centers in 6 countries in sub-Saharan Africa. We used a Bayesian mixed-effect model to assess association between ART and TB prevalence and used adaptive lasso regression to analyze risk factors for adverse TB outcomes. The study period encompassed 57,525 patient-years and 1,160 TB cases (2,017 cases/100,000 patient-years). Every 10% increase in ART uptake resulted in a 2.33% reduction in TB prevalence. Favorable TB outcomes were associated with increased time in care and early ART initiation, whereas severe immunosuppression was associated with death. These findings support integrated HIV/TB services for HIV-infected children and adults and demonstrate the association of ART uptake with decreased TB incidence in high HIV/TB settings.

Tuberculosis (TB) is an underestimated cause of death in children (*1*); it is accurately diagnosed and reported in only 45% of children with the disease (*2*). When accounting for underdetection, the World Health Organization (WHO) estimated that, in 2017, a total of 1.12 million TB cases developed in infants, children, and adolescents <14 years of age and 1.60 million cases in adolescents and young adults 15–24 years of age (*2*). WHO also estimated that TB was associated with 205,000 deaths in children, including 32,000 in HIV-infected children and adolescents; these deaths account for 13% of total TB-associated deaths in HIV-infected persons, although only 5% of HIV-infected persons are children (*2*). Children might be at increased risk for TB because they receive antiretroviral therapy (ART) at lower rates than adults. According to the Joint United Nations Programme on HIV/AIDS (*3*), only 53% of eligible children worldwide received ART in 2019.

Before ART was widely available, TB incidence and TB-related deaths were substantially higher among HIV-infected children and adolescents than among peers without HIV (*4*). Multiple studies have demonstrated declines in TB incidence among this group after ART scale-up initiatives (*5*,*6*). A meta-analysis of data from children estimated a pooled hazard ratio of 0.3 (95% CI 0.21–0.39) and declining TB risk for 2 years after ART initiation (*5*,*7*). However, TB remains a major cause of illness and death in children receiving ART (*8*).

Although risk factors for TB and adverse TB outcomes are well-documented among HIV-infected adults (*9*), risk factors among HIV-infected children and adolescents are poorly understood, particularly since the 2016 recommendations for universal ART for all HIV-infected persons (*10*). Some systematic reviews suggest that immunosuppression predicts TB incidence (*7*) among HIV-infected children and adolescents. Studies examining data sourced from a single country typically demonstrate that 1 or 2 risk factors, such as age <2 years, extrapulmonary TB, malnutrition, severe immunosuppression, WHO HIV clinical stage, or TB treatment status (*11*–*14*) can predict death among children with HIV-associated TB. Large or multinational studies of TB risk factors and outcomes among HIV-infected children and adolescents are few (*15*,*16*) and urgently needed.

Limited and conflicting data exist on outcomes among HIV-infected children and adolescents in whom TB developed before versus after ART initiation. In a multinational cohort of children from predominantly resource-limited countries, no association existed between TB outcomes and disease onset relative to ART initiation (*15*). In contrast, other studies have demonstrated lower death rates among children on ART at the time of TB diagnosis (*17*). WHO recommends that HIV-infected children and adolescents start ART as soon as possible and within <8 weeks of beginning TB treatment (*18*). Recent evidence derived from the same multinational cohort demonstrates that this recommendation was poorly implemented; only 46% of ART-naive children began treatment within <8 weeks of starting TB treatment. However, when implemented successfully, this measure was associated with favorable TB treatment outcomes (64% vs. 40%; p = 0.04) (*15*).

Data regarding TB incidence, management, and outcomes among HIV-infected children and adolescents are mostly sourced from single healthcare centers, limiting their generalizability. We analyzed these variables in the largest reported multinational study of TB in HIV-infected children and adolescents in countries in sub-Saharan Africa.

## Materials and Methods

### Strengthening the Reporting of Observational Studies in Epidemiology Statement

This study was conducted in accordance with the Strengthening the Reporting of Observational Studies in Epidemiology guidelines (*19*). Our primary objectives were to estimate longitudinal TB incidence within the context of increasing ART coverage and identify risk factors for death from TB in HIV-infected children and adolescents in various stages of ART.

### Participants

We examined TB outcomes of HIV-infected children and adolescents receiving care at 7 treatment centers (Centers of Excellence; COEs) within the Baylor International Pediatric AIDS Initiative at Texas Children’s Hospital network, spanning 6 countries: Botswana, Swaziland, Lesotho, Malawi, Tanzania (locations in Mbeya and Mwanza), and Uganda. To avoid sampling bias, we included data from all children receiving care during from January 2013 through June 2017.

### Outcomes of Interest

We used TB case and outcome definitions from WHO (*20*) (Appendix Table 1). We categorized TB outcomes as favorable (cured or treatment completed), unfavorable (death), lost to follow-up (LTFU), or transferred out of the COE.

### Data Extraction

We analayzed data from Janaury 2013 through June 2017 from the electronic medical records of HIV-infected children and adolescents <19 years of age. The deidentified data from the 7 COEs enables follow-up of individual patients for longitudinal analysis.

The duration of each COE’s study period depended on when that COE began collecting TB data using electronic medical records. Data collection began in 2013 at all COEs (except for Malawi, which began data collection in 2016) and continued through June 2017.

### Statistical Approach

We calculated the annual TB incidence for all HIV-infected children and adolescents at each COE. We modeled the TB incidence as a function of time, ART uptake, and COE. We included a random intercept for the COE, enabling us to visualize how ART uptake varied by COE and by year. We ran this model under a Bayesian framework using R with the library brms (*21*). We used a similar Bayesian mixed-effect model to determine association between isoniazid preventive therapy (IPT) use and TB incidence (*22*). Data on IPT use were available from 4 COEs: Swaziland, Tanzania-Mbeya, Tanzania-Mwanza, and Uganda. We considered children to be eligible for IPT if they were >12 months of age and had not previously received IPT.

We examined variables in bivariate analysis if >75% of data were available. We excluded some variables, such as specific anthropometrics (e.g., height, mid-upper arm circumference) and *Mycobacterium bovis* BCG vaccination status because of missing data. We considered CD4 values and viral loads if measured <60 days before or after the analytic baseline. For bivariate analysis, we used a χ^2^ test for categorical independent variables and a Wilcoxon rank-sum test for continuous independent variables. We used multinomial logistic regression to study the univariate association between age and outcome. To relax the assumption of age having a constant effect on outcome, we modeled age using natural cubic splines, using the splines package in R, with 3 knots at equally spaced quantiles. To determine age-related risk for death we calculated the instantaneous rate of change using the method of finite differences.

We used an adaptive lasso logistic regression model to examine the association between TB outcome and 13 independent variables (*23*). Adaptive lasso normalizes coefficients; therefore, no reference level is preselected. Instead, the model minimizes the influence of coefficients unassociated with the outcome; these coefficients set the reference levels for the remaining coefficients. We selected the penalty parameter to minimize deviance using leave-one-out cross-validation; we used the selected penalty to fit the final model. We included risk factors that we hypothesized, a priori, would affect TB outcomes (Appendix Table 2). We conducted the adaptive lasso procedure using library glmnet in R (*23*) and postselection inference of the selected coefficients using library selective Inference, also in R (*24*). We developed 3 models to examine associations with favorable outcomes (cure or treatment completion) against death. We examined the outcome within all participants, participants on ART at time of TB diagnosis, and participants not on ART at diagnosis. We conducted sensitivity analyses of HIV-infected children and adolescents who were LTFU or died. We developed additional models evaluating outcomes categorized by WHO as favorable (cure or treatment completion) or unfavorable (death, LTFU, or not evaluated/transferred out) (Appendix Tables 3–5).

### Ethics Statement

All clinical investigation supporting the data handling, analysis, and reporting of these findings was conducted according to the principles expressed in the Declaration of Helsinki. Approval was obtained from all necessary ethics bodies in each country (i.e., the Baylor College of Medicine Children’s Foundation or Trust, the national ethics committee in each country, and the Baylor College of Medicine Institutional Review Board).

## Results

### TB Incidence

We analyzed data on 1,160 HIV-infected children and adolescents in whom TB was diagnosed during the study period, which encompassed 57,525 patient-years. During the 4-year study period, overall TB incidence was 2,017 cases/100,000 patient-years (range 454 cases/100,000 patient-years in Botswana to 4,385 cases/100,000 patient-years in Tanzania-Mwanza). These incidences were substantially higher than those estimated by WHO for the general populations of the respective countries (Table 1).

**Table 1:** Comparison of country-specific incidence of HIV-associated TB, 2013–2017*

Country	TB incidence no. per 100,000 patient-years (95% CI)	WHO 2017 country estimates, no. per 100,000 persons (95% CI)		TB incidence/WHO 2017 TB country estimate fold difference
		TB	HIV-associated TB	
Botswana	454 (299–608)	300 (232–376)	144 (93–206)	1.5
Eswatini	2,612 (2,205–3,020)	308 (236–389)	213 (138–304)	8.5
Lesotho	3,762 (3,376–4,148)	665 (430–949)	470 (298–680)	5.6
Malawi	1,159 (791–1,528)	131 (70–210)	65 (42–93)	8.8
Tanzania-Mwanza	4,385 (3,747–5,024)	269 (127–464)	84 (54–120)	16.3
Tanzania-Mbeya	3,995 (3,498–4,492)	269 (127–464)	84 (54–120)	14.8
Uganda	656 (546–766)	201 (118–305)	80 (52–114)	3.2


*TB incidence reflects the estimated new cases of TB disease among HIV-infected children and adolescents at each HIV treatment center. HIV-associated TB incidence rate reflects the estimated rate of HIV-associated TB relative to the population as a whole, including adults and children. TB, tuberculosis; WHO, World Health Organization.

**Figure 1:**
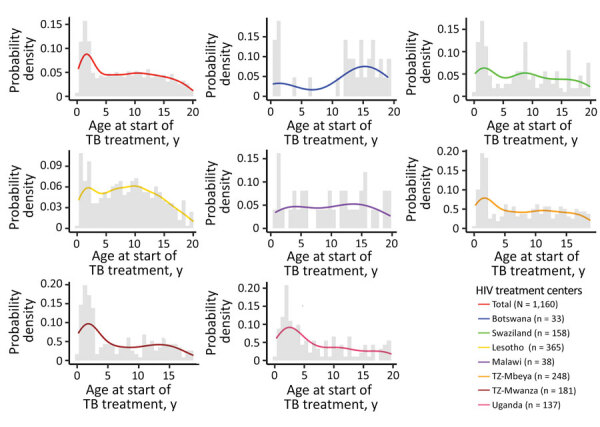
Incidence of tuberculosis (TB) among HIV-infected children and adolescents, 2013–2017. The age at start of TB treatment is plotted as a smoothed line and histogram against the probability of TB diagnosis on the basis of the prevalence of that age within the overall cohort of HIV-infected children and adolescents. The data are presented combined and stratified by HIV treatment center.

**Figure 2:**
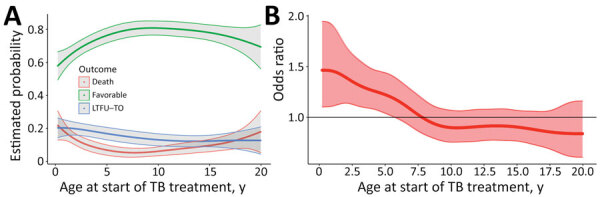
Probablities of specific outcomes for TB in HIV-infected children and adolescents, 2013–2017. A) Probability (with 95% CIs) of outcomes stratified by age at start of TB treatment. B) Instantaneous odds ratios for death at each age. The odds ratio reflects the change in odds of death according to age at start of TB treatment. LTFU-TO, lost to follow-up or transferred out; TB, tuberculosis.

The age distribution of the cohort was similar across sites. TB incidence was highest among infants and children <5 years of age but was elevated among school-aged children, a trend that persisted into early and late adolescence (Figure 1). Increasing age was associated with more favorable outcomes. Children <7 years of age had a higher risk for death than school-aged children (i.e. 8–10 years of age) and adolescents (i.e., 11–19 years of age) (Figure 2, panels A, B). Of patients receiving TB treatment, 32% had TB infection confirmed by bacteriologic testing, usually GeneXpert (Cepheid, https://www.cepheid.com); this percentage excludes children at the Lesotho COE because it had incomplete data. Confirmation rates ranged from 24% for infants and children <2 years of age to 51% for adolescents 10–19 years of age.

**Figure 3:**
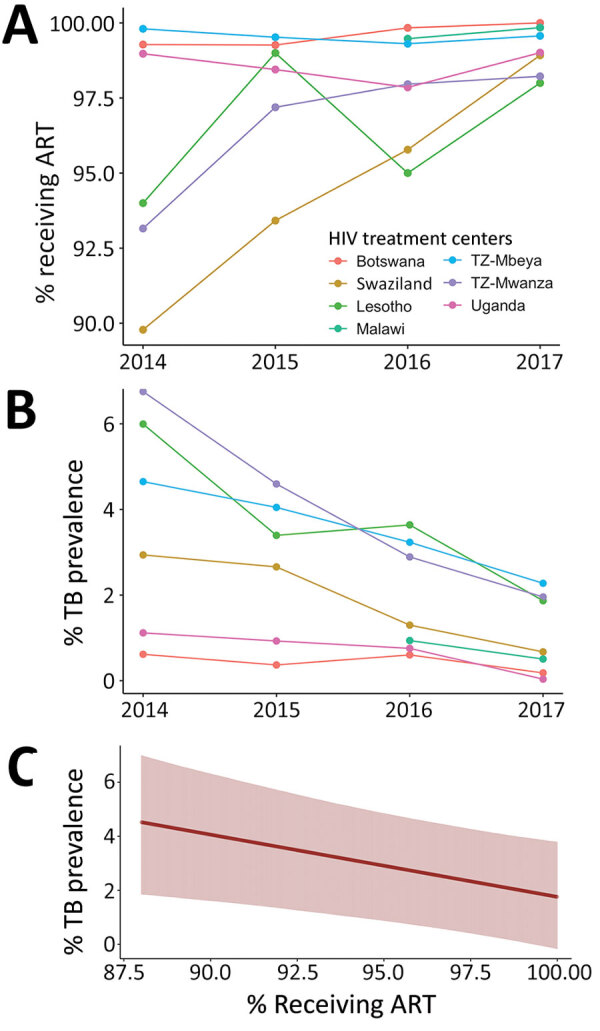
ART use and TB prevalence in HIV-infected children and adolescents, 2013–2017. A) Annual percentage of the cohort at each HIV treatment center receiving ART. B) Annual percentage of the cohort at each treatment center in whom TB was diagnosed. C) Declining TB prevalence with increase in ART uptake, averaged across all treatment centers in the study period. ART, antiretroviral therapy; TB, tuberculosis.

Throughout the study, rates of ART administration increased and the prevalence of TB declined at most sites (Figure 3, panels A, B). For every 10% increase in the number of HIV-infected children and adolescents who received ART, the overall prevalence of TB in the clinical network decreased 2.33% (95% credible interval 0.58%–4.4%) (Figure 3, panel C).

We also observed an increase in the number of eligible children starting IPT. Average rates of IPT use across all COEs increased from 8.68% in 2014 to 27.5% in 2017. When we limited our analysis to the 4 COEs with available IPT data, we observed no effect on TB prevalence (0.4% increase, 95% credible interval –0.3% to 1.0%) associated with each 10% increase in the number of HIV-infected children and adolescents receiving IPT.

### TB Outcomes and Risk Factor Analysis

Most children and adolescents had favorable outcomes: across all sites, 75% (95% CI 67%–87%) of patients, including those who were LTFU or had transferred out, had favorable outcomes (Table 2). On average, children with favorable TB outcomes had received care at the clinics nearly a year longer than children who died (p<0.01). Ten percent (95% CI 5%–15%) of HIV-infected children and adolescents with TB died. If we assumed all HIV-infected children and adolescents who were LTFU died, the death ratio would increase to 13% (95% CI 6%–20%).

**Table 2:** Bivariate analysis of associations with TB treatment outcomes in HIV-infected children and adolescents, 2013–2017*

Variable	Outcome				p value†
	Total	Completed TB treatment or cured	Died	Lost to follow up or transferred out	
Sex					0.98
F	591 (50.95)	438 (51.11)	61 (50.41)	92 (50.55)	
M	569 (49.05)	419 (48.89)	60 (49.59)	90 (49.45)	
HIV treatment center					<0.01
Botswana	33 (2.84)	31 (3.62)	0	2 (1.10)	
Eswatini	365 (32.39)	302 (35.24)	15 (12.40)	48 (26.37)	
Lesotho	38 (4.99)	33 (3.85)	3 (2.48)	2 (1.10)	
Malawi	158 (13.62)	134 (15.64)	15 (12.40)	9 (4.95)	
Tanzania–Mbeya	248 (21.38)	160 (18.67)	41 (33.88)	47 (25.82)	
Tanzania–Mwanza	181 (15.60)	106 (12.37)	18 (14.88)	57 (31.32)	
Uganda	137 (11.81)	91 (10.62)	29 (23.97)	17 (9.34)	
Site of TB					<0.01
Pulmonary TB	997 (88.39)	760 (90.15)	87 (78.38)	150 (86.21)	
Extrapulmonary TB	131 (11.61)	83 (9.85)	24 (21.62)	24 (13.79)	
TB treatment category					0.08
Newly treated TB patient	1,007 (90.56)	764 (91.50)	96 (91.43)	147 (85.47)	
Previously treated TB patient	105 (9.44)	71 (8.50)	9 (8.57)	25 (14.53)	
TB drug resistance testing					<0.01
Not tested	837 (75.61)	612 (73.38)	82 (78.10)	143 (90.51)	
Not detected	258 (23.31)	220 (26.38)	23 (21.70)	15 (9.04)	
Mono-resistance detected	6 (0.54)	2 (0.24)	1 (0.94)	3 (1.81)	
Multidrug-resistance detected	6 (0.54)	0	0	5 (3.01)	
ART regimen at start of TB treatment					0.41
Efavirenz-based	225 (40.61)	177 (42.34)	20 (31.75)	28 (38.36)	
Nevirapine-based	166 (29.96)	130 (31.10)	16 (25.40)	20 (27.40)	
Lopinavir-based	134 (24.19)	91 (21.77)	23 (36.51)	20 (27.40)	
Atazanavir-based	17 (3.07)	12 (2.87)	3 (4.76)	2 (2.74)	
Other	9 (1.62)	6 (1.44)	1 (1.59)	2 (2.74)	
Azidothymidine + lamivudine + abacavir	3 (0.54)	2 (0.48)	0	1 (1.37)	
ART relative to start of TB treatment					<0.01
On ART >6 mos	421 (34.59)	327 (36.01)	42 (34.71)	52 (27.66)	
On ART <8 wks after TB treatment	396 (32.54)	315 (34.69)	26 (21.49)	55 (29.26)	
On ART <6 mos	276 (22.68)	190 (20.93)	38 (31.40)	48 (25.53)	
Never started ART	97 (7.97)	58 (6.39)	12 (9.92)	27 (14.36)	
On ART >8 wks after TB treatment	27 (2.22)	18 (1.98)	3 (2.48)	6 (3.19)	
Immune status at start of TB treatment					<0.01
Nonadvanced	468 (55.19)	387 (58.46)	15 (22.73)	66 (55.00)	
Severe	252 (29.72)	174 (26.28)	41 (62.12)	37 (30.83)	
Advanced	128 (15.09)	101 (15.26)	10 (15.15)	17 (14.17)	
Mean time receiving care before TB diagnosis, d		635.5	363.3	342	<0.01
Mean time on ART before TB diagnosis, d		697.6	624	508.6	0.12

*Values are no (%) except as indicated. ART, antiretroviral therapy; TB, tuberculosis. †χ^2^ test.

We used bivariate analysis to identify factors associated with TB outcome (Table 2). Extrapulmonary disease increased the odds of death (p<0.01). The death ratio for patients who had previously been treated for TB was similar to the death ratio for patients who had not, even if we assumed all patients who were LTFU died. HIV-infected children and adolescents who had engaged in facility-based HIV care for more time were more likely to have favorable outcomes (p<0.01).

**Figure 4:**
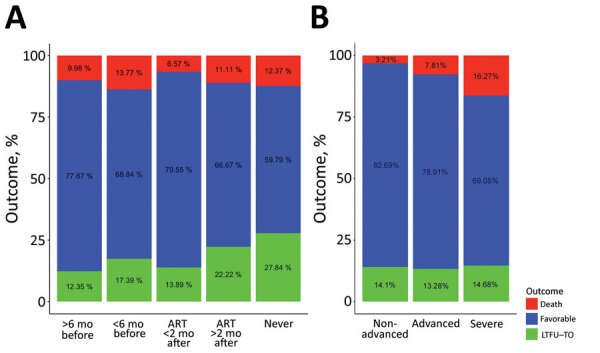
Bivariate analyses of factors relating to TB treatment outcome in HIV-infected children and adolescents, 2013–2017. A) ART treatment category: received ART >6 months or <6 months before TB diagnosis or started on ART <2 months or >2 months after TB diagnosis. B) Immune status. Advanced immunosuppression was define as a CD4 percentage of <25% in children <5 years of age or CD4 count <200 cells/mm^3^ in children >5 years of age. ART, antiretroviral therapy; TB, tuberculosis.

HIV-infected children and adolescents who never started ART were less likely than those in all other groups to have a favorable outcome (Figure 4, panel A). HIV-infected children and adolescents who began ART during the 6 months before the start of their TB treatment had the highest death ratio (14%); children who had never been on ART but began it within 8 weeks of TB diagnosis had the lowest death ratio (8%). Immune status was highly predictive of death (Figure 4, panel B). HIV-infected children and adolescents with severe immunosuppression had >5-fold increased odds of death compared with those without immune suppression (16% vs. 3%; p<0.01); we defined severe immunosuppression as a CD4 percentage of <25% in children <5 years of age or CD4 count <200 cells/mm^3^ in children >5 years of age.

We developed 3 multivariate models to comprehensively examine associations with favorable outcomes against death, considering a patient’s history of ART. The model comparing all 1,029 patients considered 11 factors (Table 3). This model demonstrated the influence of immune status at the time of TB diagnosis, showing that HIV-infected children and adolescents without immune suppression (CD4 percentage >30% in children <5 years of age or CD4 count >350 cells/mm^3^ in children >5 years of age) at TB diagnosis had a 58% lower odds of death (odds ratio [OR] 0.42 [95% CI 0.13–0.94]; p = 0.04) than children with advanced immune suppression. In addition, patients who had never been on ART but received it <8 weeks after TB diagnosis had a 59% lower odds of death than those who received ART >8 weeks after TB diagnosis (OR 0.41 [95% CI 0.14–0.60]; p<0.01). The multivariate analysis did not demonstrate an increased odds of death for HIV-infected children and adolescents who started ART in the 6 months before TB diagnosis.

**Table 3:** Predictors of favorable TB outcomes against death in HIV-infected children and adolescents, 2013–2017*

Variable	Odds ratio (95% CI)	p value
Country		
Swaziland, Lesotho, Malawi, Tanzania-Mbeya, Tanzania-Mwanza, and Uganda	Referent	
Botswana	0.09 (0.00–22.83)	0.1811
TB drug resistance		
Not tested, multidrug resistance, and not detected	Referent	
Monoresistance	18.11 (0.00–31,381.00)	0.4642
ART category		
On ART >6 mos before TB treatment, on ART <6 mos before TB treatment, and on ART <8 wks after TB treatment	Referent	
Never on ART	3.38 (0.21–20.02)	0.2168
On ART >8 wks after starting TB treatment	0.41 (0.14–0.60)	0.0051†
Immune status		
Advanced	Referent	
Not advanced	0.42 (0.13–0.94)	0.0412†
Severe	1.88 (0.70–4.80)	0.1233
Each increasing WHO stage	3.64 (2.50–7.17)	<0.001‡

*Variables included were sex, COE, site of TB, TB treatment category, TB drug resistance, age at ATT initiation, days in care at COE, immune status, ART TB days, ART category, and World Health Organization stage; all elements under each variable are described in Table 2. Variables excluded were ART regimen pre-ATT initiation, and total ART drugs pre-ATT treatment. ART, antiretroviral therapy; ATT, anti-TB therapy; COE, Center of Excellence; TB, tuberculosis. †Significant result (p<0.05). ‡Significant result (p<0.01).

The model comparing the 597 patients who had received ART before TB diagnosis considered 13 factors (Table 4). Children with severe immunosuppression at TB diagnosis had a 4 times higher odds of death than children with advanced immune suppression (OR 4.29 [95% CI 1.23–29.28]; p = 0.03). A patient’s odds of death increased with each advance in WHO stage at TB diagnosis (OR 2.18 [95% CI 1.91–5.98]; p<0.01).

**Table 4:** Predictors of favorable TB outcomes against death in HIV-infected children and adolescents on ART at TB diagnosis, 2013–2017

Variable	Odds ratio (95% CI)	p value
Country		
Swaziland, Lesotho, Malawi, Tanzania-Mbeya, Tanzania-Mwanza, and Uganda	Referent	
Botswana	0.22 (0.01–1.90)	0.0858
TB drug resistance		
Not tested, multidrug resistance, not detected	Referent	
Mono INH resistance	23.64 (0.00–28,630.46)	0.3917
Immune status		
Advanced	Referent	
Not advanced	0.59 (0.12–5.17)	0.2767
Severe	4.29 (1.23–29.28)	0.0294†
Each increasing WHO stage	2.18 (1.91–5.98)	0.001†

*Variables included were sex, COE, site of TB, TB treatment category, TB drug resistance, age at ATT initiation, days in care at COE, ART regimen pre-ATT initiation, immune status, ART TB days, total ART drugs pre-ATT treatment, ART category, and World Health Organization stage. ART, antiretroviral therapy; ATT, anti-TB therapy; COE, Center of Excellence; TB, tuberculosis. †Significant result (p<0.05).

The final model of patients who had never received ART comprised 391 patients with favorable outcomes and 41 patients who died; this model was intractable and did not converge. Results evaluating associations with programmatic outcomes were similar to the results described in the previous paragraphs (Appendix Tables 3–5).

## Discussion

TB is the leading cause of death in HIV-infected persons (*2*). This multicountry study of TB in HIV-infected children and adolescents revealed high TB incidences that greatly exceeded estimated population level TB incidences of all individual countries represented by the cohort. Consistent with recent systematic reviews and meta-analyses (*7*), our evidence demonstrates that although ART significantly reduces the prevalence of TB in HIV-infected children and adolescents, the risk for TB remains elevated even among a population with excellent ART coverage. TB-related deaths decreased in adults during 1996–2011, when ART use increased in various countries (*25*). Although we cannot ascribe causality between increasing ART coverage and declining TB prevalence, this association is notable given the very high initial ART coverage in our study. Increasing ART uptake >90% was associated with ongoing declines in TB incidence, suggesting that the Joint United Nations Programme on HIV/AIDS 95-95-95 targets for HIV might also reduce TB incidence. Likewise, HIV-infected children and adolescents with favorable TB outcomes had received care for nearly a year longer, on average, than children who died. These findings highlight the importance of early HIV diagnosis, prompt treatment, and patient retention in HIV-infected children and adolescents.

Of HIV-infected children and adolescents with TB, 32% had a confirmed TB diagnosis; the rate of TB confirmation increased with age. This high rate of disease confirmation is consistent with prior studies, which have found that HIV infection does not significantly reduce the likelihood of disease confirmation in children (*26*–*28*). However, other multinational cohorts have reported lower rates of disease confirmation in HIV-infected children and adolescents, a discrepancy that might reflect the greater testing capacity at the COEs (*15*). Most HIV clinics treating children in sub-Saharan Africa do not have the capacity to collect TB specimens in children (*29*). Confirmatory diagnostic testing remains a challenge more broadly across low and middle-income countries in sub-Saharan Africa; furthermore, current testing strategies are invasive and extremely uncomfortable, necessitating the development of child-friendly diagnostic tools.

Within the cohort, HIV-infected children and adolescents <5 years of age had the greatest risk for TB, a finding consistent with existing literature (*14*). Likewise, children in this age range were more likely to die (*4*). However, TB incidence did not decline significantly among school-age children <10 years of age, an age group in HIV-negative cohorts that has a significantly reduced risk for TB (*30*). This analysis, like others (*31*), suggests that HIV reduces the protective effect of age on TB risk.

Overall, 75% of HIV-infected children and adolescents in this cohort had favorable treatment outcomes, whereas 10% died. Existing literature estimates that in sub-Saharan Africa, 16% of HIV-infected children and adolescents who are LTFU die, regardless of TB status (*32*). Because 4% of patients in our study were LTFU, the death rate of our cohort might be closer to 10%–13%. Recent evidence estimates a cumulative all-cause death rate of 3% at 3 months, 5% at 6 months, 6% at 12 months, and 7% at 24 months after ART initiation in HIV-infected children and adolescents in sub-Saharan Africa (*33*).

Approximately 60% of HIV-infected children and adolescents have severe immunosuppression at TB diagnosis (*13*). In contrast, 33% of children in this cohort had advanced or severe immunosuppression at TB diagnosis. Severe immunosuppression was associated with up to a 4-fold higher risk for death than advanced immunosuppression. Similarly, advanced WHO stage (noted before TB diagnosis) was associated with a 2-fold increased risk for death. This finding highlights the need for scale-up of HIV identification and treatment methods, such as family index testing, provider-initiated testing and counseling, Test and Start models (*10*), and prompt ART initiation. Public health officials must further evaluate the care of children and adolescents with advanced HIV to develop strategies that promote survival (*34*).

The bivariate and multivariate analyses demonstrate a dramatic reduction of death in children who started ART within <8 weeks after TB diagnosis. Multivariate analysis shows that starting ART within <8 weeks after beginning TB treatment was associated with a 59% reduction in death compared with children on ART before their TB diagnosis or beginning it >8 weeks after starting TB treatment. Furthermore, children who never initiated ART had a >3 times higher risk for death than those who were on ART before their TB diagnosis or began ART >8 weeks after starting TB treatment. We need more data to ascertain whether ART can further reduce HIV-associated TB death in children when initiated within <2 weeks after TB treatment. Most HIV-infected children and adolescents with TB have paucibacillary disease, which can inhibit confirmatory TB testing and might reduce children’s risk for immune reconstitution syndrome after starting ART. Furthermore, children have lower risk for adverse reactions to TB treatment and ART than adults. Thus, early simultaneous initiation of ART and TB treatment might be a safer treatment strategy in children. Starting ART at the same time as TB treatment might reduce the number of HIV-infected children and adolescents with TB who are LTFU before starting ART. This promising treatment strategy should be evaluated in controlled studies.

Among HIV-infected children and adolescents who were not on ART at TB diagnosis, 85% started ART within <8 weeks of beginning TB treatment, confirming that this intervention is attainable in a setting with high HIV/TB prevalence. Children who started ART <8 weeks after TB treatment had the lowest odds of death, even lower than those of children on ART at TB diagnosis. We hypothesize that many children who develop TB while on ART are not virologically surpressed and are therefore more likely to die from this disease. The bivariate analysis, but not the multivariate analysis, indicated an increased odds of death among children who began ART <6 months before TB diagnosis. This finding suggests that HIV-infected children and adolescents with unmasking TB-immune reconstitution syndrome, an exaggerated inflammatory manifestation of TB during early ART, might have an increased odds of death. Prospective studies evaluating time from TB diagnosis to ART initiation has benefitted the treatment of adults with HIV (*35*). Similar prospective trials are needed to inform treatment of HIV-infected children and adolescents.

This study has some limitations. Because we used data from clinical settings, missing data precluded analysis of some variables. Nevertheless, this evidence is representative of well-managed clinics in countries in sub-Saharan Africa with consistent access to diagnostic technology and ART medications. We identified an association between ART and TB prevalence but cumulative IPT coverage also increased over time across a subset of COEs. We did not observe an association between IPT uptake and TB prevalence. This lack of association might have been caused by sample size limitations, inconsistent IPT availability, or heterogeneity of IPT guidelines; therefore, we cannot draw strong conclusions about the effects of IPT on TB prevalence. Because only a subset of COEs provided data on IPT, we did not include it in the TB outcomes models. We could not compare annual incidence of TB at the clinic level with national trends because of recent changes in WHO’s estimation methods (*36*). Last, as with all retrospective analysis, the possibility exists for inaccurate entry of clinical data. We attempted to limit this source of error through manual data checks.

The strong association between immune suppression at TB diagnosis and death highlights the importance of early TB case detection and ART initiation among HIV-infected children and adolescents. Furthermore, we found a strong association between favorable TB outcome and increased length of time in care. These associations emphasize the importance of early HIV case detection and prompt ART initiation. Finally, we found an association between increased ART coverage and decreased TB incidence, as well as ART initiation within <8 weeks after starting TB treatment and favorable outcomes. Collectively, these findings support the continued need to promote policies and implement practices that fully integrate optimal HIV and TB treatment in countries with high burdens of these diseases.

Appendix Definitions and outcomes for HIV-associated tuberculosis among children and adolescents in high HIV/TB settings.
