# Supplementary material for: Tuberculosis among Children and Adolescents at HIV Treatment Centers in Sub-Saharan Africa
Source: Emerg Infect Dis. 2020 Dec;26(12):2933–43. doi: 10.3201/eid2612.202245 (PMC7706926; doi:10.3201/eid2612.202245)
Supplement: Appendix — Definitions and outcomes for HIV-associated tuberculosis among children and adolescents in high HIV/TB settings. [file 20-2245-Techapp-s1.pdf]

Article DOI: https://doi.org/10.3201/eid2612.202245

# Tuberculosis Among Children and Adolescents at HIV Treatment Centers in Sub-Saharan Africa

## Appendix

**Appendix Table 1.** World Health Organization 2013 tuberculosis case definitions

| Term | Definition |
|---|---|
| Bacteriologically confirmed | A bacteriologically confirmed tuberculosis (TB) case is one from whom a biological specimen is positive by smear microscopy, culture or World Health Organization Recommended Diagnostic (such as Xpert MTB/RIF). All such cases should be notified, regardless of whether TB treatment has started. |
| Clinically diagnosed | A clinically diagnosed TB case is one who does not fulfill the criteria for bacteriological confirmation but has been diagnosed with active TB by a clinician or other medical practitioner who has decided to give the patient a full course of TB treatment. |
| Additional classifications | Bacteriologically confirmed or clinically diagnosed cases of TB are also classified according to: i) anatomical site of disease; ii) history of previous treatment; iii) drug resistance; iv) HIV status. |
| Site of disease | Pulmonary tuberculosis refers to any bacteriologically confirmed or clinically diagnosed case of TB involving the lung parenchyma or the tracheobronchial tree. Miliary TB is classified as pulmonary TB because there are lesions in the lungs. Tuberculous intrathoracic lymphadenopathy (mediastinal and/or hilar) or tuberculous pleural effusion, without radiographic abnormalities in the lungs, constitutes a case of extrapulmonary TB. A patient with both pulmonary and extrapulmonary TB should be classified as a case of pulmonary TB. Extrapulmonary tuberculosis refers to any bacteriologically confirmed or clinically diagnosed case of TB involving organs other than the lungs, e.g. pleura, lymph nodes, abdomen, genitourinary tract, skin, joints and bones, meninges. |
| New TB patients | New patients have never been treated for TB or have taken TB drugs for <1 month. |
| Previous treatment | Classifications on the basis on history of previous TB treatment are slightly different from those previously published. They focus only on history of previous treatment and are independent of bacteriological confirmation or site of disease. New patients have never been treated for TB or have taken TB drugs for <1 month. Previously treated patients have received ≥1 month of TB drugs in the past. |
| HIV status | HIV-positive TB patient refers to any bacteriologically confirmed or clinically diagnosed case of TB who has a positive result from HIV testing conducted at the time of TB diagnosis or other documented evidence of enrolment in HIV care, such as enrollment in the pre-antiretroviral therapy (ART) register or in the ART register once ART has been started. HIV-negative TB patient refers to any bacteriologically confirmed or clinically diagnosed case of TB who has a negative result from HIV testing conducted at the time of TB diagnosis. Any HIV-negative TB patient subsequently found to be HIV-positive should be reclassified accordingly. An HIV status unknown TB patient refers to any bacteriologically confirmed or clinically diagnosed case of TB who has no result of HIV testing and no other documented evidence of enrollment in HIV care. If the patient's HIV status is subsequently determined, he or she should be reclassified accordingly. |
| Drug resistance | Cases are classified in categories on the basis of drug susceptibility testing of clinical isolates confirmed to be *Mycobacterium tuberculosis*: i) Monoresistance: resistance to one first-line TB drug only; ii) Polydrug resistance: resistance to >1 first-line TB drug (other than both isoniazid and rifampicin); iii) Multidrug resistance: resistance to at least both isoniazid and rifampicin; iv) Extensive drug resistance: resistance to any fluoroquinolone and to ≥1 of 3 second-line injectable drugs (capreomycin, kanamycin and amikain), in addition to multidrug resistance. |
| Cured | A pulmonary TB patient with bacteriologically confirmed TB at the beginning of treatment who was smear- or culture-negative in the last month of treatment and on ≥1 previous occasion. |
| Treatment completed | A TB patient who completed treatment without evidence of failure but with no record to show that sputum smear or culture results in the last month of treatment and on ≥1 previous occasion were negative, either because tests were not done or because results are unavailable. |
| Treatment failed | A TB patient whose sputum smear or culture is positive at month 5 or later during treatment. |
| Died | A TB patient who dies for any reason before starting or during the course of treatment. |
| Lost to follow-up | A TB patient who did not start treatment or whose treatment was interrupted for ≥2 consecutive months. |
| Not evaluated | A TB patient for whom no treatment outcome is assigned. This includes cases "transferred out" to another treatment unit as well as cases for whom the treatment outcome is unknown to the reporting unit. |

**Appendix Table 2.** Factors analyzed with respect to TB biologic and programmatic outcomes*

| Factor | Variable type | Definition(s) |
|---|---|---|
| Sex | Categorical | F<br>M |
| COE | Categorical | Botswana<br>Swaziland<br>Lesotho<br>Malawi<br>Tanzania-Mbeya<br>Tanzania-Mwanza<br>Uganda |
| Site of TB | Categorical | Pulmonary TB<br>Extrapulmonary TB |
| TB diagnostic category | Categorical | Bacteriologically confirmed TB<br>Unconfirmed (clinically diagnosed) TB |
| TB treatment category | Categorical | Newly treated TB patient<br>Previously treated TB patient |
| Age at start of TB treatment | Continuous | Years |
| Calendar days in care at COE (at start of TB treatment) | Continuous | Days |
| Calendar days between ART initiation and start of TB treatment | Continuous | Days |
| TB drug resistance (considers all test available to COE clinicians) | Categorical | Not tested<br>Not detected<br>Mono-resistance<br>Multidrug-resistance |
| ART regimen at start of TB treatment | Categorical | Efavirenz-based<br>Nevirapine-based<br>Lopinavir-based<br>Atazanavir-based<br>Other<br>Azidothymidine + Lamivudine + Abacavir |
| Total ART drugs used before starting TB treatment | Continuous | Number of different individual ART drugs patient has been exposed to during care and treatment |
| World Health Organization clinical stage before TB diagnosis | Categorical | I and II combined<br>III<br>IV |
| ART timeline relative to start of TB treatment | Categorical | Received ART for >6 mo before starting TB treatment<br>Initiated ART ≤2 mo of starting TB treatment<br>Received ART for ≤6 mo before starting TB treatment<br>Never initiated ART<br>Initiated ART >2 mo after starting TB treatment |
| Immune status at start of TB treatment† | Categorical | Children <5 y of age<br>Nonadvanced = CD4 ≥30%<br>Severe = CD4 <25%<br>Advanced = CD4 <30%<br>Children ≥ 5 y of age<br>Nonadvanced = CD4 count >350 cells/$mm^3$<br>Severe = CD4 count <200 cells/$mm^3$<br>Advanced = CD4 count 200–350 cells/$mm^3$ |

*ART, antiretroviral therapy; COE, Center of Excellence; TB, tuberculosis.
†Derived by the average of the past 2 CD4 absolute count or CD4 percentages (whichever applicable according to age) before starting TB treatment. If this data was missing, the value was derived by the first CD4 absolute count or CD4 percentage ≤60 days before or after starting TB treatment.

**Appendix Table 3.** Predictors of TB outcome of HIV-infected children and adolescents, 2013–2017*

| Variable | Odds Ratio (95% CI) | p value |
|---|---|---|
| Country | | |
| Swaziland and Uganda | 1 (NA) | NA |
| Botswana | 0.34 (0.08–1.61) | 0.11 |
| Lesotho | 1.34 (0.52–3.31) | 0.22 |
| Malawi | 0.76 (0.23–333.33) | 0.67 |
| Tanzania-Mbeya | 0.73 (0.40–2.64) | 0.34 |
| Tanzania-Mwanza | 2.09 (1.31–3.98) | 0.01† |
| TB treatment category | | |
| Previously treated | 1 (NA) | NA |
| Newly treated TB patient | 0.44 (0.22–0.70) | 0.01‡ |
| TB drug resistance | | |
| Not tested | 1 (NA) | NA |
| Mono resistance | 10.42 (0.00–59855.52) | 0.66 |
| Multidrug resistance | 312.99 (0.00–33460573.72) | 0.34 |
| Not detected | 0.52 (0.29–0.84) | 0.02‡ |
| ART category*)* | | |
| On ART >6 mo after starting TB treatment | 1 (NA) | NA |
| Never on ART | 15.98 (4.55–70.81) | <0.01† |
| On ART ≤2 mo after starting TB treatment | 0.67 (0.25–1.25) | 0.12 |
| On ART ≤6 mo before starting TB treatment | 1.13 (0.24–2.00) | 0.50 |
| On ART >2 mo after starting TB treatment | 2.03 (0.52–60.01) | 0.17 |
| Immune status | | |
| Nonadvanced and advanced | 1 (NA) | NA |
| Severe | 1.84 (1.39–2.97) | <0.01† |
| Each increasing World Health Organization stage | 2.21 (1.74–3.39) | <0.01† |

*Favorable outcomes defined as cure or completion and unfavorable as death, lost to follow-up or not evaluated ART, antiretroviral therapy; TB, tuberculosis.
†statistically significant association with unfavorable treatment outcome
‡statistically significant association with favorable treatment outcome

**Appendix Table 4.** Predictors of TB outcome among HIV-infected children and adolescents on ART at TB diagnosis, 2013–2017*

| Variable | Odds Ratio (95% CI) | p value |
|---|---|---|
| Country | | |
| Swaziland, Lesotho, Malawi, Uganda | 1 (NA) | NA |
| Botswana | 0.24 (0.03–1.05) | 0.0531 |
| Tanzania-Mbeya | 0.59 (0.19–2.02) | 0.1759 |
| Tanzania-Mwanza | 4.04 (2.40–8.97) | 0† |
| TB treatment category | | |
| Previously treated patients | 1 (NA) | NA |
| Newly treated TB patients | 0.40 (0.11–0.57) | 0.0035‡ |
| TB drug resistance | | |
| Not tested, not detected | 1 (NA) | NA |
| Mono resistance | 1.69 (0.00–44.34) | 0.8704 |
| Multidrug resistance | 319.65 (0.00–8229767.53) | 0.2522 |
| ART regimen before starting TB treatment | | |
| Efavirenz, Lopinavir, Nevirapine | 1 (NA) | NA |
| Azidothymidine + Lamivudine + Abacavir | 0.15 (0.00–2223.87) | 0.4454 |
| Atazanavir/ritonavir | 0.42 (0.05–6.48) | 0.2286 |
| Other | 3.63 (0.68–19.70) | 0.0871 |
| Immune status | | |
| Nonadvanced, advanced | 1 (NA) | NA |
| Severe | 3.81 (2.66–9.50) | 0† |
| Each increasing World Health Organization stage | 1.72 (1.39–3.04) | 0.0028† |

*Favorable outcomes defined as cure or completion and unfavorable as death, lost to follow-up, or not evaluated. ART, antiretroviral therapy; TB, tuberculosis.
†statistically significant association with unfavorable treatment outcome
‡statistically significant association with favorable treatment outcome

**Appendix Table 5.** Predictors of TB outcome among HIV-infected children and adolescents who had never taken ART, 2013–2017*

| Variable | Odds Ratio (95% CI) | p value |
|---|---|---|
| Country | | |
| Lesotho, Malawi, Tanzania-Mbeya, Tanzania-Mwanza, Uganda | 1 (NA) | NA |
| Botswana | 0.17 (0.00–10000.00) | 0.38 |
| Swaziland | 0.49 (0.06–1.56) | 0.11 |
| TB treatment category | | |
| Previously treated patients | 1 (NA) | NA |
| Newly treated TB patients | 0.46 (0.08–3.17) | 0.19 |
| Increasing age, y | 0.57 (0.34–0.70) | <0.01‡ |
| TB drug resistance | | |
| Not tested, multidrug resistance, mono resistance | 1 (NA) | NA |
| Not detected | 0.77 (0.26–10.93) | 0.49 |
| ART category | | |
| Started ART >2 mo after starting TB treatment | 1 (NA) | NA |
| Never on ART | 12.55 (12.57–66.76) | <0.01† |
| Started ART≤2 mo after starting TB treatment | 0.35 (0.10–0.89) | 0.04‡ |
| Each increasing World Health Organization stage | 1.25 (0.44–2.13) | 0.36 |

*Favorable outcomes defined as cure or completion and unfavorable as death, lost to follow-up, or not evaluated. ART, antiretroviral therapy; TB, tuberculosis.
†statistically significant association with unfavorable treatment outcome
‡statistically significant association with favorable treatment outcome
